# On the Treatment of the Bowel Affections of Infancy and Childhood Incident to the Summer Months

**Published:** 1878-09

**Authors:** P. A. Allaire

**Affiliations:** Aurora, Ill.


"*On the Treatment of the Bowel Affections of Infancy and Childhood Incident to the Summer Months.*"

In the August Number of the Journal and Examiner appears an article by Dr. DeLaskie Miller, on the above-named subject, really valuable, and embodying more common-sense in the treatment and hygiene than can be found elsewhere. Yet it seems to me that the most important factor in the management of these cases has been omitted. However important in the causation of the diseases under discussion may be a vitiated atmosphere and improper diet, it is quite certain that without a high temperature in addition, these diseases would not occur. With a pure atmosphere and good diet we have the same class of cases in the country. Heat is surely there the important factor in the production of "summer complaint." To reduce the temperature, then is the primary indication. Each little patient should be wrapped in a wet sheet extending from the axillæ to the knees, and kept in a *recumbent position.* The head should be freely bathed with cool water if hot, and no food given for the first twenty-four hours, only a little cold water if it can be had pure. Thus the temperature of the body should be *reduced to the natural standard and kept so, as nearly as possible.* If we could give our patients pure, cool air to breathe in addition, little would be left for the medication which Dr. Miller has so well summed up.

After the first day or two the activity of the fever usually subsides, but the wet sheet should be continued until the evacuations become healthy, and then replaced with a flannel band to support the abdomen. I append a typical case as the disease appears here.

J. F., one year old, a strong, well-nourished boy, fed with cow's milk from a nursing bottle. Aug. 10th. Feverish and restless; did not take food as well as usual. Aug. 11th. Increase of fever, bowels loose, ate part of an apple; p. m., vomiting and purging. At six (evening) I was called in, and found the child in its first convulsion, head hot, feet and hands cool, pulse 140. He had vomited five or six times during the previous four hours, and purged as often, the evacuations being watery with mucus and spots of blood on the diapers.

Treatment.—Free application of cold water to the head, the body wrapped in a cool wet sheet from arm pits to the knees. Hot cloths to the feet and legs. The child recovered from the convulsion and became sensible in about half an hour. No recurrence. At 9 p. m. child feverish and restless, pulse 115. Gave a mixture containing six drops of paregoric and six drops of syrup of ipecac every hour until quieted. Also kept on the wet pack, and kept the head cool by bathing. No food. Aug. 12th. a. m. Child slept three or four hours, and has had three evacuations of the bowels; no vomiting; is still a little feverish, pulse 108; wants food. I directed 50 Gms. boiled milk every three hours, and a tablespoonful of cool water every half hour, if thirsty. Continue the wet sheet and give paregoric and ipecac every two hours. Evening. He had two evacuations to-day, the last one small and healthy-looking; skin cool and moist; head cool; playful. I directed a flannel bandage to be placed around the abdomen and strict attention to be paid to the diet. No medicine. Dismissed.

P. A. Allaire,
Aurora, Ill.

---

We are pleased to note that the adoption of the metric system in the pages of this journal has already produced most desirable results. Our exchanges have given us due credit as the first of American medical journals to take this decided step; and a few have not hesitated to follow in the same path. Many of the practitioners in Chicago have already begun to employ the system exclusively in the writing of their prescriptions, and all of our really high-class druggists have prepared themselves to compound medicines so ordered.

We have received many acknowledgments of the benefits derived from the change, and have every reason to congratulate ourselves upon the result.

We have received from the American Metric Bureau a number of slips convenient for carrying in a physician's call book, which explain the metric system so that it becomes not only intelligible but of practical value. We will be pleased to distribute them to physicians who will enquire for the same at the Library rooms, No. 188 South Clark st.

